# Gram scale preparation of clozapine *N*-oxide (CNO), a synthetic small molecule actuator for muscarinic acetylcholine DREADDs

**DOI:** 10.1016/j.mex.2018.03.003

**Published:** 2018-03-23

**Authors:** Phillip L. van der Peet, Christian Gunawan, Alaa Abdul-Ridha, Sherie Ma, Daniel J. Scott, Andrew L. Gundlach, Ross A.D. Bathgate, Jonathan M. White, Spencer J. Williams

**Affiliations:** a School of Chemistry and Bio21 Molecular Science and Biotechnology Institute, The University of Melbourne, Victoria 3010, Australia; b The Florey Institute of Neuroscience and Mental Health, Parkville, Victoria 3052, Australia; c Florey Department of Neuroscience and Mental Health, The University of Melbourne, Victoria 3010, Australia; d Department of Biochemistry and Molecular Biology, The University of Melbourne, Victoria 3010 Australia

**Keywords:** Preparation and application of clozapine N-oxide (CNO), G-protein-coupled receptor (GPCR), Chemical-genetics, Chemical synthesis

## Abstract

Chemogenetics uses engineered proteins that are controlled by small molecule actuators, allowing *in vivo* functional studies of proteins with temporal and dose control, and include Designer Receptors Exclusively Activated by Designer Drugs (DREADDs). One major class of DREADDs are mutated muscarinic receptors that are unresponsive to acetylcholine, and are activated by administration of clozapine *N*-oxide (CNO). However, CNO is available in only small amounts and large scale studies involving animals and multiple cohorts are prohibitively expensive for many investigators. The precursor, clozapine, is also expensive when purchased from specialist suppliers. Here we report:

• A simple extraction method of clozapine from commercial tablets;
• A simple preparation of CNO from clozapine, and for the first time its single-crystal X-ray structure; and
• That the CNO prepared by this method specifically activates the DREADD receptor hM3Dq *in vivo*.

This method provides large quantities of CNO suitable for large-scale DREADD applications that is identical to commercial material.

## Graphical abstract

**Figure:**
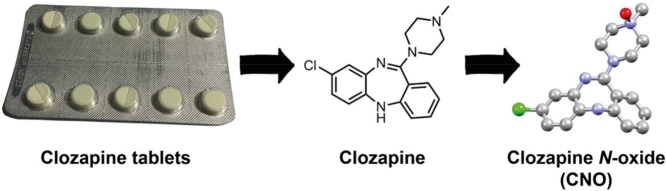

**Specifications Table**

**Table:** 

Subject area	*Pharmacology, Toxicology and Pharmaceutical Science*
More specific subject area	*Chemogenetics*
Method name	Preparation and application of clozapine *N*-oxide (CNO)
Name and reference of original method	D. Bender, M. Holschbach, G. Stocklin, Synthesis of n.c.a. carbon-11 labelled clozapine and its major metabolite clozapine-*N*-oxide and comparison of their biodistribution in mice, Nucl. Med. Biol. 21 (1994) 921-5, doi:10.1016/0969-8051(94)90080-9.
Resource availability	*Not applicable*

## Method details

### Overview

DREADD (designer receptors exclusively activated by designer drugs) technology is a chemical-genetic approach that posits the development of mutated G-protein-coupled receptors (GPCRs) that no longer respond to a receptor-specific drug or the endogenous ligands, but which respond exclusively to a designer drug that is otherwise inert and inactive [[1], [2]]. Mutant DREADD receptors are expressed within cells or organisms and provide a powerful way to exert transient and repeatable control of receptor function. DREADDs were initially developed for the muscarinic acetylcholine receptor family [3], but have since been applied to a range of GPCRs, including β_2_-adrenergic receptor [4], κ-opioid receptors [5] and 5-HT_2A_ receptors [6]. A range of mutant muscarinic acetylcholine receptors have been developed [2], including axonally-targeted activating and silencing subtypes [7], as well as arrestin-biased receptors [8]. Muscarinic DREADD receptors contain two mutations of conserved orthosteric site residues that cause loss of responsiveness to their native ligand acetylcholine, and respond specifically upon treatment with the small molecule clozapine *N*-oxide (CNO) [3]. While initially it was believed that CNO was the activating ligand, recent studies suggest that CNO does not enter the brain; rather metabolic conversion yields clozapine, which readily enters the brain and occupies DREADDs expressed within the central nervous system [9]. CNO therefore may function as a precursor to deliver ‘sub-threshold' clozapine that selectively activate DREADDs. Nonetheless, CNO does provide activating effects and remains an important reagent for *in vitro* and *in vivo* studies of brain function, provided that well-controlled experimental design is used to control for effects of clozapine beyond those at DREADDs [10].

CNO is commercially available, but its high cost has limited its application for *in vivo* animal studies where multiple animal cohorts and extended dosing regimens require sizeable (multigram) quantities of CNO [[11], [12]]. Furthermore, because of redox cycling of CNO to clozapine that can confound DREADD effects [9], clozapine is also required in significant quantities for control experiments [13]. Consequently, we required a scalable approach that could provide large quantities of clozapine and CNO.

The preparation of CNO has been described several times. The most common approach involves the oxidation of clozapine with mCPBA, which provides CNO in yields of 86–94% [[14], [15], [16]]. Körber reported oxidation using hydrogen peroxide was lower yielding than mCPBA [15]. Herein we report a new synthetic approach allowing the synthesis of gram-scale quantities of CNO using the inexpensive oxidizing agent, Oxone^®^ (2KHSO_5_·KHSO_4_·K_2_SO_4_).

We have previously used CNO synthesized using the method reported here to study the effects of chemogenetic activation of the hindbrain *nucleus incertus* (NI), which promoted cortical arousal, locomotor activity, and risk-assessment behavior [12]. Importantly, no effects on cortical activity or behavior were observed in CNO-injected control rats expressing the mCherry fluorophore only, suggesting that there are no off-target effects from CNO-derived clozapine. The NI is largely GABAergic and is the primary source of the neuropeptide, relaxin-3 (RLN3), in rat [[17], [18], [19]], mouse [[20], [21]] and macaque [22]. RLN3 has been demonstrated to robustly increase food intake in rats [[23], [24], [25]], therefore in this study, we used a simple home-cage food and water intake assay to test the effects of synthesized CNO on food and water intake in rats expressing hM3Dq-mCherry or mCherry-only in the NI.

## Experimental

### Extraction of clozapine from clopine tablets

One hundred Clopine tablets (Pfizer, each tablet 100 mg of clozapine) were crushed and added to 250 ml of acetone and stirred at 50 °C for 60 min. The mixture was filtered through a pad of Celite, rinsed with acetone and the volume of filtrate was reduced under vacuum. Petroleum spirit was added to the concentrated filtrate until crystals were observed. The mixture was cooled on ice and filtered to afford clozapine (9.8 g, 98%) as yellow crystals; m.p. 183–184 °C; lit. [26] 182–184 °C; ^1^H NMR (500 MHz, CDCl_3_) *δ* 2.34 (s, 3H), 2.50 (brs, 4H), 3.47 (brs, 4H), 4.88 (s, 1H), 6.60 (d, *J* = 8.4 Hz, 1H), 6.82 (dd, *J* = 8.4, 1.2 Hz, 2H), 7.01 (t, *J* = 7.2 Hz, 1H), 7.06 (d, *J* = 2.4 Hz, 1H), 7.25-7.29 (m, 2H); ^13^C NMR (125 MHz, CDCl_3_) *δ* 46.3, 47.4, 55.1, 120.1, 120.2, 123.1, 123.2, 123.6, 126.9, 129.2, 130.4, 132.0, 140.5, 141.9, 152.8, 162.9; HRMS (ESI) calcd for C_18_H_20_ClN_4_ [M + H]^+^ 327.1371. Found 327.1372.

### Preparation of clozapine-N-oxide

Oxone monopersulfate (4.29 g, 6.68 mmol) was added to a solution of clozapine (4.37 g, 13.4 mmol) and NaHCO_3_ (2.25 g, 26.7 mmol) in MeOH (25 ml) and H_2_O (5 ml) at r.t. After stirring at r.t. for 2 h, the reaction mixture was concentrated and purified by silica gel column chromatography (60% CH_2_Cl_2_/MeOH) to afford an orange oil. The oil was crystallized from EtOH to afford clozapine *N*-oxide as the EtOH solvate (4.46 g, 97%). m.p. 235–237 °C, lit. [14] 224 °C (from EtOH); lit. [16] 248–249 °C (from EtOAc); lit. [15] 199–206 °C; ^1^H NMR (500 MHz, CD_3_OD) *δ* 3.17 (br d, *J* = 11.5 Hz, 2H), 3.24 (s, 3H), 3.60 (br t, *J* = 11.0 Hz, 2H), 3.74 (br t, *J* = 11.0 Hz, 2H), 3.86 (brs, 2H), 6.81 (d, *J* = 8.0 Hz, 1H), 6.86 (dd, *J* = 2.5, 8.5 Hz, 1H), 6.97 (d, *J* = 2.0 Hz, 1H), 7.00 (dd, *J* = 1.0, 8.0 Hz, 1H), 7.05 (dt, *J* = 1.0, 8.5 Hz, 1H), 7.31 (dd, *J* = 1.5, 8.0 Hz, 1H), 7.36 (dt, *J* 1.5, 7.5 Hz, 1H); ^13^C NMR (125 MHz, CD_3_OD) *δ* 43.2, 60.1, 65.8, 121.4, 121.5, 123.9, 124.2, 124.9, 127.3, 129.6, 131.1, 133.7, 142.8, 143.2, 155.5, 164.1; HRMS (ESI) calcd for C_18_H_20_ClN_4_O [M + H]^+^ 343.1320. Found 343.1322.

### High performance liquid chromatography

The HPLC system consisted of Agilent 1100 series RP-HPLC equipped with a diode array detector at 275 nm. The column was SGE ProteCol C18 H 125 250 × 4.6 mm, 5 μm particle size, 120 Å pore size. The compounds were eluted with a gradient of 0–95% B over 25 min, where A was 100% H_2_O + 0.1% TFA and B was 100% CH_3_CN + 0.1% TFA.

### Crystallography

Intensity data were collected with an Oxford Diffraction Sapphire CCD diffractometer using Cu-Kα radiation (graphite crystal monochromator λ = 1.54184), the temperature during data collection was maintained at 130.0(1) K using an Oxford Cryosystems cooling device. The structure was solved by direct methods and difference Fourier synthesis [27]. A thermal ellipsoid plot was generated using the program ORTEP-3 [28] integrated within the WINGX [29] suite of programs.

Crystal data for CNO. C_18_H_19_N_4_OCl. (CH_3_CH_2_OH) *M* = 388.89, *T* = 130.0(2) K, λ = 1.5418, Orthorhombic, space group Pbca *a* *=* 12.9985(2), *b* *=* 16.9693(3), *c* *=* 17.2455(3) *Å, V* *=* 3803.93(11) *Å^3^*, Z = 8, D_c_ = 1.358 mg M^−3^ μ(Cu-Kα) 1.967 mm^−1^, F(000) = 1648, crystal size 0.3 × 0.18 × 0.15 mm. 21252 reflections measured, 3961 independent reflections (R_int_ = 0.0378) the final R was 0.0369 [I > 2σ(I), 3402 data] and *w*R(F^2^) was 0.1011 (all data). The data has been deposited at the Cambridge Crystallographic Data Centre: CCDC 1554367.

### Cell culture

Chinese hamster ovary (CHO) FlpIn cells stably expressing the human M_1_ muscarinic acetylcholine receptor (mAChR) harboring the DREADD mutations (Y106C and A196G) were obtained from The Monash Institute of Pharmaceutical Sciences. Cells were maintained in DMEM (Dulbecco’s modified Eagle’s medium) containing 10% foetal bovine serum (FBS) and 0.6 mg/ml hygromycin B at 37 °C in a humidified incubator containing 5% CO_2_.

### Extracellular signal-regulated kinase 1/2 phosphorylation (pERK1/2) assay

Assays to measure M_1_ DREADD-mediated stimulation of ERK1/2 phosphorylation were performed using the Alpha Screen based Sure-Fire kit (TGR Biosciences), following the manufacturer’s instructions. Briefly, FlpIn CHO cells stably expressing the M_1_ DREADD were seeded into 96-well culture plates at 40,000 cells per well and allowed to adhere. Cells were then rinsed with phosphate-buffered saline and maintained in serum-free media overnight at 37 °C, 5% CO_2_. The following day, cells were stimulated with agonist. Initial pERK1/2 time course experiments were performed to determine the time of maximal ERK1/2 phosphorylation for CNO and clozapine (found to be 5 min for both ligands). The time of peak agonist response was then used for the establishment of concentration-response curves. In all experiments, 10% (v/v) foetal bovine serum (FBS) was used as positive control of pERK1/2. The reaction was terminated by removal of media and addition of lysis buffer. Samples were processed according to kit instructions. The fluorescence signal was measured using a POLARstar Omega plate reader (BMG Labtech). Data were normalized to the maximum response elicited by 10% (v/v) FBS at 5 min.

### Animal experiments

Animal experiments, including all surgical procedures, were conducted with the approval of The Florey Institute of Neuroscience and Mental Health Animal Ethics Committee and according to ethical guidelines of the National Health and Medical Research Council of Australia. All efforts were made to minimize stress prior to experimentation and the number of rats used. Male Sprague-Dawley rats (250–300 g; Animal Resources Centre; Canning Vale, WA, Australia) were single-housed under ambient conditions (21 °C) and maintained on a 12 h light:dark cycle (lights on at 07:00) with free access to food and water. Rats were acclimatized to the institute animal facility for 1 week prior to any experimentation.

### Generation of AAV-sCAG-(hM3Dq)-mCherry-WPRE viral vector

The pAAV-sCAG-mCherry-WPRE vector was prepared using the Life Technologies Gateway cloning system, as described [[12], [30], [31]]. For assembly of pAAV-sCAG-mCherry-WPRE a three plasmid recombination reaction was performed using 2 μl LR Clonase™ II (Life Technologies, Mulgrave, VIC, Australia) in a 10 μl reaction (also containing 20 fmol pAAV-Gateway attLR1-R2 destination plasmid, 10 fmol pENTR-attL1-sCAG-attL4, 10 fmol pENTR-attR4-mCherry-attR3, 10 fmol pENTR-attL3-WPRE-L2). The reaction was incubated overnight (16 h) at r.t. and then was treated with 1 μl of proteinase K for 15 min at 37 °C before transformation into chemically-competent Stbl3 *E. coli* cells. pAAV-sCAG-hM3Dq-mCherry-WPRE, Entry (pENTR) plasmids and pAAV-Gateway destination plasmid were a gift from Dr Melanie White (Institute of Molecular and Cell Biology, Agency for Science, Technology and Research (A*STAR), Singapore).

Both the pAAV-sCAG-hM3Dq-mCherry-WPRE and pAAV-sCAG-mCherry-WPRE were separately packaged into a rAAV mosaic serotype 1/2 capsid using the packaging plasmids pDPI and pDPII, as described [32]. Vectors were harvested and purified using iodixanol gradients and viral titer, as determined by genomic copies (gc) per ml, was examined using quantitative polymerase chain reaction (qPCR). Reactions were conducted in a total volume of 25 μl containing 12.5 μl of SYBR Green Master Mix, 1 μl of forward primer (10 μM), 1 μl of reverse primer (10 μM), 5 μl of MgCl_2_ (25 mM), 0.5 μl of water and 5 μl of either viral sample or standard. qPCR amplification was completed using an ABI Prism 7500HT wSequence Detection device (Applied Biosystems, Mulgrave, VIC, Australia) with conditions of 10 min at 95 °C followed by 40 cycles of denaturation at 95 °C of 15 s, annealing at 67 °C for 25 s, and extension/measurement at 72 °C for 25 s. Previously described primers specific for the WPRE transcriptional element were used [33] to amplify viral genomes. Plasmid pAAV-sCAG-mCherry-WPRE was used to produce the qPCR standard curve.

### Stereotaxic injection of viral vectors

Rats (n = 12) were stereotaxically injected with pAAV-sCAG-hM3Dq-mCherry-WPRE or pAAV-sCAG-mCherry-WPRE into the hindbrain NI, as described [12]. Rats were anesthetized by inhalation of 4% isoflurane delivered in air into an enclosed chamber and placed into a stereotaxic frame (Kopf Instruments) with anesthesia maintained by 2–3% isoflurane delivered at 200 ml/min in air using a rat anesthetic mask (Kopf Instruments). Rats were injected bilaterally with 300–400 nl of AAV1/2-sCAG-hM3Dq-mCherry (5.2 × 10^7^ gc; n = 8) or AAV1/2-sCAG-mCherry (1.6 × 10^8^ gc; n = 4). Viral vectors were pressure injected into the NI using a pulled glass pipette attached to a 1 μl syringe (10–20 μm) at AP −2.6 mm; ML ±0.1 mm; DV −6.4 mm from lambda; incisor bar −12.5 mm) at 0.2 μl/min. To minimize reflux, the pipette remained in place for a further 10 min, was raised 1 mm and left in place for a further 1 min before being slowly withdrawn completely. Rats were injected at least >4 weeks prior to testing.

### In vivo test of food and water intake in rats

On the day of use, CNO was dissolved in sterile saline at 1 mg/ml and administered by intraperitoneal (i.p.) injection in rats at 1 or 3 mg/kg, or rats were injected with an equivalent volume of sterile saline. For behavioral testing, rats received an i.p. injection of sterile saline on Day 1 and placed back into their home cage with access to pre-weighed food and water, and consumption was recorded 4 h post-injection. Testing was repeated on Day 2, where rats received an i.p. injection of CNO at 1 or 3 mg/kg.

Food and water intake following CNO injection were normalized to intake following saline injection, where a value of 1 represents equivalent intake. Unpaired *t*-tests were used to calculate statistical differences between groups.

### Viral transduction analysis

Sections from viral vector-injected rats were processed for relaxin-3 immunostaining in relation to innate hM3Dq-mCherry fluorescence. Rats were deeply anaesthetized with pentobarbitone (100 mg/kg, i.p.) and transcardially perfused with 300 ml of ice-cold 0.1 M phosphate-buffered saline (PBS) followed by 400 ml of 4% formaldehyde in PBS. The brains were dissected and submerged in 30% sucrose in PBS for 48 h at 4 °C. Coronal sections (30 μm) through the rostrocaudal extent of the brainstem, through the NI, were collected free-floating into PBS. Sections were incubated in blocking buffer (10% v/v NGS in PBS with 0.1% Triton-X) for 1 h with agitation at r.t. Sections were then incubated in PBS containing mouse anti-relaxin-3 (1:5, HK4-144-10; [[18], [34]], 2% NGS, and 0.1% Triton-X for 18 h at r.t. Sections were washed 3 × 10 min followed by incubation in 1:500 dilutions of Alexa Fluor-488-conjugated donkey anti-mouse (Jackson ImmunoResearch, West Grove, PA, USA) in PBS for 1 h at r.t. Sections were washed 3 × 5 min, slide-mounted and coverslipped with Fluoromount-G (Southern Biotech, Birmingham, AL, USA). Stitched image tiles were acquired with a Zeiss 780 Confocal microscope, as described [12].

## Method details

### Chemical synthesis

Clozapine itself is commercially available, but from typical suppliers is expensive (e.g. Sigma-Aldrich, $1910/g). Instead, clozapine can be isolated from prescription tablets (100 tablets each 100 mg containing 10 g clozapine; total cost $140, $14/g, 1% of the cost from Sigma-Aldrich) by a simple extraction process. Thus Clopine tablets (100 mg/tablet) were crushed in a mortar and pestle, then stirred with acetone. Filtration to remove excipient, followed by evaporation produced a solid which was recrystallized from acetone/petroleum spirits.

**Fig. 1:**
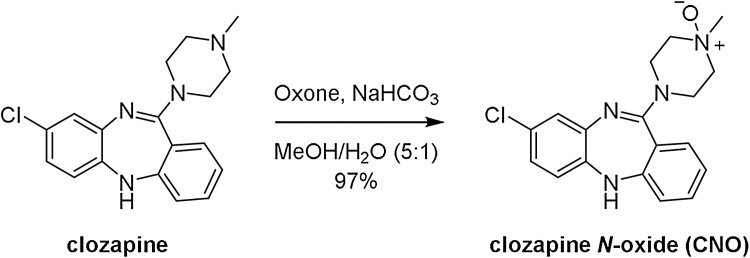
Oxidation of clozapine to clozapine *N*-oxide (CNO) using Oxone.

We initially attempted the oxidation of clozapine with mCPBA [16]. While the transformation was successful, yields were modest and the reaction mixture suffered from extensive coloration which proved difficult to remove from the isolated product by chromatography, decolorization with charcoal and/or recrystallization from methanol, EtOH, or CH_2_Cl_2_/petroleum spirits. To overcome these problems we surveyed a range of oxidation conditions, including H_2_O_2_ in refluxing EtOH [35], ammonium molybdate/H_2_O_2_ [36] and Oxone^®^ [[37], [38], [39]]. The most promising results were obtained using Oxone^®^ in MeOH, which provided a smooth transformation of clozapine to CNO (Fig. 1). Like oxidations performed with mCPBA, extensive coloration of the reaction mixture was obtained, but in this case the color was effectively removed by silica gel chromatography and subsequent crystallization from EtOH. The extracted clozapine and synthesized CNO were assessed for purity by high performance liquid chromatography (hplc; see Supporting information). Conditions were established that allowed resolution of clozapine from CNO. The extracted clozapine was determined to be 99.5% purity, and the recrystallized CNO prepared using the described method was determined to be 99.1% purity, using a photo diode array detector at 275 nm with no detectable impurity of clozapine.

### Characterization

**Fig. 2:**
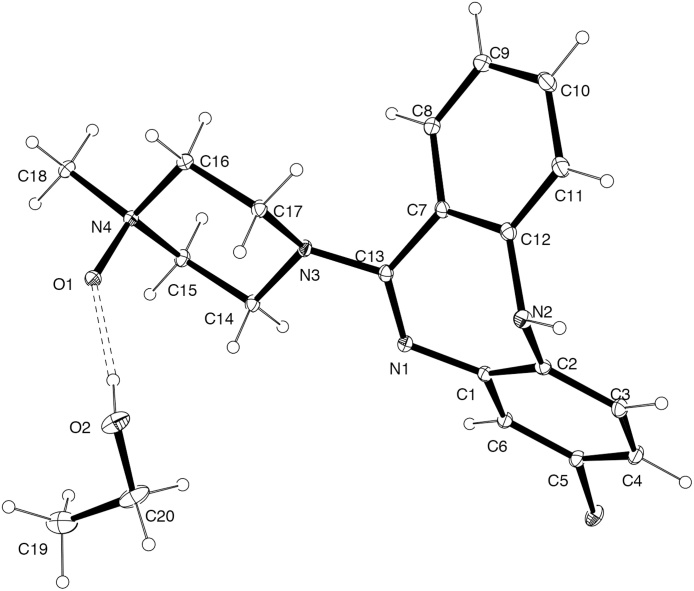
ORTEP representation of the molecular structure of CNO. EtOH, determined by single-crystal X-ray crystallography. Ellipsoids are at the 30% probability level.

Commercial samples of CNO are typically available as solvates with methanol or ethanol. CNO can be readily crystallized from EtOH, and this procedure provided large crystals suitable for X-ray crystallographic analysis, revealing a single molecule of EtOH as a solvate (Fig. 2).

### Biological activity

**Fig. 3:**
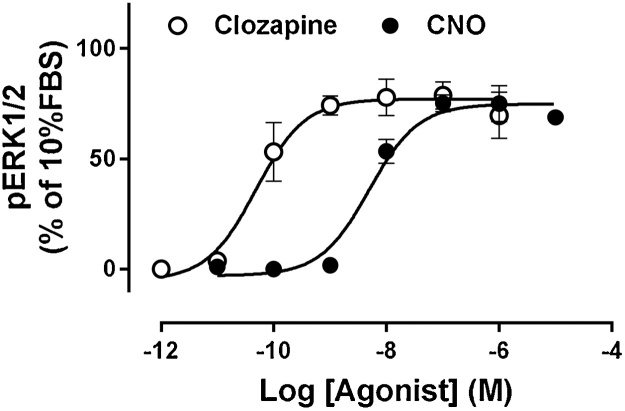
Activation profiles of CNO and clozapine at the M_1_ DREADD. Data points represent the mean ± SEM of three independent experiments performed in duplicate.

In studies to confirm the biological activity of the CNO synthesized herein and the extracted clozapine, we measured their ability to activate the M_1_ DREADD in cells using the ERK1/2 phosphorylation assay as a signaling endpoint linked to this receptor. CNO and clozapine produced potent and efficacious responses (pEC_50_ 8.31 ± 0.12 and 10.32 ± 0.18; E_max_ 74.8 ± 2.8 and 77.0 ± 3.7, respectively, Fig. 3). The potencies observed are comparable to the potencies of commercial CNO and clozapine (Sigma Aldrich) reported with this cell line (pEC_50_ 8.50 ± 0.13 and 9.68 ± 0.28, respectively) [40].

### In vivo activity

**Fig. 4:**
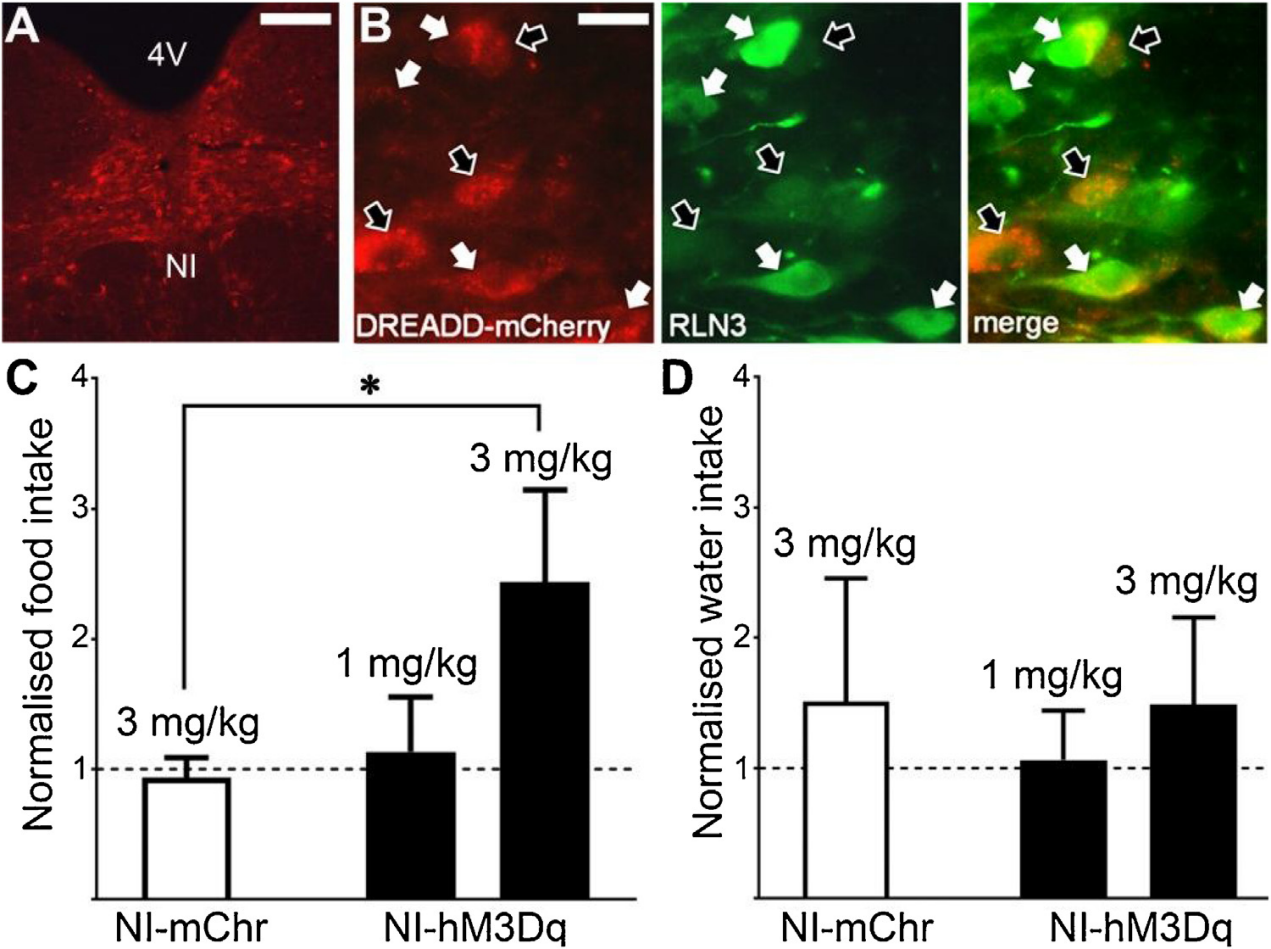
**(A)** hM3Dq-mCherry fluorescence in the NI of an NI-hM3Dq rat indicates transduction of NI neurons located midline below the 4th ventricle (4 V). **(B)** hM3Dq-mCherry is expressed in RLN3 (white arrows) and non-RLN3 (black arrows) neurons. **(C)** Food and **(D)** water intake of control NI-mCherry and NI-hM3Dq injected with 1 or 3 mg/kg CNO. Scale bars (A) 100 μm and (B) 20 μm.

In studies to confirm the biological activity of the CNO synthesized as described *in vivo*, we examined the effects of chemogenetic activation of the NI in rats. The modified Gq-coupled human muscarinic receptor, hM3Dq [41], fused to mCherry fluorescent protein was expressed in NI neurons using an adeno-associated viral vector expression system with a strong, chicken β-actin (CAG) promoter – AAV1/2-sCAG-hM3Dq-mCherry. An AAV1/2-sCAG-mCherry vector was used as a control. Microinjection of this viral vector into the NI resulted in hM3Dq expression (hereafter referred to as NI-hM3Dq) in NI soma and proximal processes, as indicated by the presence of fluorescent mCherry puncta (Fig. 4A,B). The control AAV1/2-sCAG-mCherry vector (hereafter referred to as NI-mCherry), in contrast, produced profuse red cytoplasmic fluorescence in NI cells (data not shown), as described [12]. We have shown that chemogenetic NI activation in NI-hM3Dq rats leads to cortical arousal, decreased sleep, increased locomotor activity, and increased risk-assessment behavior, all of which are consistent with increased general arousal [12]. Furthermore, these studies also demonstrated that hM3Dq activation by CNO *in vitro* resulted in long-lasting depolarization of NI neurons with action potentials, and that peripheral injection of CNO (3 mg/kg) significantly increased immunostaining for c-Fos, an immediate early gene marker of neuronal activation, in NI-hM3Dq rats, compared to control NI-mCherry rats [12]. These data confirm the functional activation of NI by CNO activation of hM3Dq in vivo and a lack of effect of CNO in control NI-mCherry rats.

The NI is the primary source of the neuropeptide, relaxin-3 (RLN3), which has been demonstrated to robustly induce feeding following cerebroventricular infusion in rats [[23], [24], [25]]. In NI-hM3Dq rats, we observed transduction of RLN3 *and* non-RLN3 neurons (i.e. presence and absence of RLN3 immunofluorescence; Fig. 4B). Thus, our hypothesis was that chemogenetic activation of the NI would promote feeding.

Control NI-mCherry rats injected with 3 mg/kg CNO exhibited equivalent food intake to that following saline injection (Fig. 4A). NI-hM3Dq rats injected with 3 mg/kg, but not 1 mg/kg, exhibited significantly increased (∼2.5-fold) food intake compared to NI-mCherry control rats (Fig. 4C; t_9_ = 2.48, *P <* 0.05). Water intake in both NI-mCherry and NI-hM3Dq rats was variable and not significantly affected by CNO (Fig. 4D). These data indicate that synthesized CNO is biologically active and activates hM3Dq *in vivo*.

## Conclusions

Increasing use of muscarinic acetylcholine receptor DREADDs has led to a growing demand for CNO, but the high cost from most commercial vendors has created difficulties for large-scale deployment of the technology [11]. This work reports simple access to clozapine from commercially available (pharmacy) tablets, and its conversion to CNO by oxidation with Oxone. Based on raw material costs (clozapine is obtained at approx. $14/g from tablets; reagent costs are minimal), CNO can be produced in a 97% yield at a similar price, excluding labor costs. By comparison, commercial prices for CNO from representative suppliers range from $53,000/g (Sigma-Aldrich), $24,000/g (Santa Cruz Biotech), $6700/g (Hello Bio). The present route with some proprietary modifications allowing removal of methanol has been implemented by AMT Pty Ltd (Melbourne, Australia), and commercial material produced by this method is now available through AK Scientific for $420/g (product code: AMTA056), representing a major reduction in the cost of this important reagent. It is hoped that this work will support the continuing development of DREADD technology and further insights into brain function and related behaviors.
