# Supplementary material for: Gram scale preparation of clozapine N-oxide (CNO), a synthetic small molecule actuator for muscarinic acetylcholine DREADDs
Source: MethodsX. 2018 Mar 23;5:257–67. doi: 10.1016/j.mex.2018.03.003 (PMC6053635; doi:10.1016/j.mex.2018.03.003)
Supplement: Supplementary file 1 [file mmc1.docx]

**SUPPORTING INFORMATION**

**Gram scale preparation of clozapine *N*-oxide (CNO), a synthetic small molecule actuator for muscarinic acetylcholine DREADDs**

Phillip L. van der Peet, Christian Gunawan, Alaa Abdul-Ridha, Sherie Ma, Daniel J. Scott , Andrew L. Gundlach, Ross A.D. Bathgate, Jonathan M. White, Spencer J. Williams

Contents

[NMR spectra 2](#_Toc484588781)

[Clozapine 2](#_Toc484588782)

[Clozapine *N*-oxide (CNO) 3](#_Toc484588783)

[Analytical HPLC traces for clozapine, clozapine N-oxide, and a mixture thereof 5](#_Toc484588784)

[1. Recrystallized clozapine; purity 99.5% 5](#_Toc484588785)

[2. Recrystallized clozapine N-oxide; purity 99.1% 5](#_Toc484588786)

[3. A 9:1 mixture of clozapine/clozapine N-oxide 7](#_Toc484588787)

# NMR spectra

## Clozapine

^1^H NMR (CDCl_3_)





^13^C NMR (CDCl_3_)





## Clozapine *N*-oxide (CNO)

^1^H NMR (CD_3_OD)





^13^C NMR (CD_3_OD)





# Analytical HPLC traces for clozapine, clozapine N-oxide, and a mixture thereof

## 1. Recrystallized clozapine; purity 99.5%

| **#** | **Time** | **Area** | **Height** | **Width** | **Area%** | **Symmetry** |
| --- | --- | --- | --- | --- | --- | --- |
| 1 | 10.201 | 24226.5 2095 | 0.2029 | 99.513 | 1.186 |  |
| 2 | 10.637 | 22.9 | 2.9 | 0.1092 | 0.094 | 0.56 |
| 3 | 15.52 | 11.6 | 1.7 | 0.1023 | 0.048 | 0.977 |
| 4 | 23.075 | 25.6 | 10.9 | 0.0394 | 0.105 | 0.878 |
| 5 | 23.167 | 58.5 | 6.6 | 0.1238 | 0.240 | 0.287 |

## 2. Recrystallized clozapine N-oxide; purity 99.1%

| **#** | **Time** | **Area** | **Height** | **Width** | **Area%** | **Symmetry** |
| --- | --- | --- | --- | --- | --- | --- |
| 1 | 10.301 | 87.6 | 14.4 | 0.0892 | 0.445 | 1.707 |
| 2 | 10.565 | 19483.4 | 1881.4 | 0.1788 | 99.059 | 1.166 |
| 3 | 15.528 | 11.1 | 1.6 | 0.1029 | 0.056 | 1.034 |
| 4 | 23.075 | 26.3 | 11.1 | 0.0375 | 0.134 | 0.878 |
| 5 | 23.166 | 60.1 | 6.7 | 0.1245 | 0.306 | 0.284 |

## 3. A 9:1 mixture of clozapine/clozapine N-oxide

| **#** | **Time** | **Area** | **Height** | **Width** | **Area%** | **Symmetry** |
| --- | --- | --- | --- | --- | --- | --- |
| 1 | 10.207 | 20821.9 1948.7 | 0.1867 | 81.963 | 1.146 |  |
| 2 | 10.612 | 4486.8 | 718.2 | 0.0949 | 17.662 | 1.135 |
| 3 | 15.521 | 11.3 | 1.6 | 0.1033 | 0.045 | 1.029 |
| 4 | 23.075 | 24.3 | 10.9 | 0.038 | 0.096 | 0.751 |
| 5 | 23.167 | 59.8 | 6.6 | 0.126 | 0.235 | 0.284 |
